# Current landscape of tumor-derived exosomal ncRNAs in glioma progression, detection, and drug resistance

**DOI:** 10.1038/s41419-021-04430-z

**Published:** 2021-12-09

**Authors:** Xiao He, Yiwei Qi, Xian Zhang, Xiaojin Liu, Xingbo Li, Sihan Li, Yiping Wu, Qi Zhang

**Affiliations:** 1 Department of Plastic Surgery, Tongji Hospital, Tongji Medical College, Huazhong University of Science and Technology, Wuhan, China; 2 Department of Neurosurgery, Tongji Hospital, Tongji Medical College, Huazhong University of Science and Technology, Wuhan, China; 3 Ningxia Key Laboratory of Cerebrocranial Diseases, Incubation Base of the National Key Laboratory, Ningxia Medical University, Yinchuan, China

**Keywords:** Cancer genomics, CNS cancer, Non-coding RNAs

## Abstract

Glioma is the most common and fatal tumor of the central nervous system in humans. Despite advances in surgery, radiotherapy, and chemotherapeutic agents, glioma still has a poor prognosis. The tumor microenvironment (TME) of glioma is of highly complex heterogeneity, which relies on a network-based communication between glioma cells and other stromal cell types. Exosomes are the most common type of naturally occurring extracellular vesicles, ranging in size from 40 to 160 nm, and can serve as carriers for proteins, RNAs, and other biologically active molecules. Recent evidence has shown that glioma-derived exosomes (GDEs) can be integrally detected in the local tissue and circulatory blood samples, and also can be transferred to recipient cells to mediate transmission of genetic information. Non-coding RNAs (ncRNAs) mainly including microRNA, long non-coding RNA, and circular RNA, account for a large portion of the human transcriptome. A broad range of ncRNAs encapsulated in GDEs is reported to exert regulatory functions in various pathophysiological processes of glioma. Herein, this review summarizes the latest findings on the fundamental roles of GDE ncRNAs that have been implicated in glioma behaviors, immunological regulation, diagnosis potential, and treatment resistance, as well as the current limitations and perspectives. Undoubtedly, a thorough understanding of this area will provide comprehensive insights into GDE-based clinical applications for combating gliomas.

## Facts

- Exosomes, especially GDEs, have been associated with the tumorigenesis, proliferation, colonization, and metastasis of glioma.
- NcRNAs are enriched and stabilized in exosomes and have attracted extensive attention due to their regulatory functions in the exchange of gene information in local and distal niches.
- GDE ncRNAs can be nonrandomly absorbed by heterologous and homologous cells to affect post-transcriptional genetic regulation, leading to behavioral changes characterized by tumorigenesis, tumor growth, invasion, and metastasis.
- GDE ncRNAs can be applied to the differential diagnosis, monitoring of post-surgical glioma progression, and even the prediction of the patient response to personalized therapies, such as vaccines, antibody drugs, and chemoradiotherapy.

## Open questions

- Whether the content and abundance of exosomes will change accordingly during different stages of glioma occurrence and development?
- What is the association between the expression of GDE ncRNAs and glioma genotyping?
- How to accurately determine which cell type-derived exosome and which GDE ncRNA dominates the influence of tumor progress at particular staging and typing of glioma?
- How do these tumor-derived exosomal ncRNAs affect the treatment resistance of glioma?

## Introduction

**Fig. 1:**
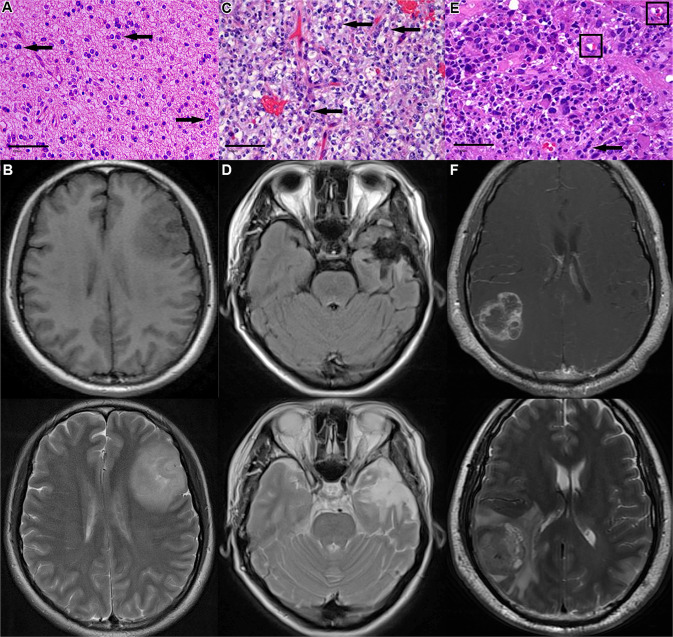
Histology characteristics and MRI images of diffuse low-grade glioma and high-grade glioma. **A** Diffuse low-grade astrocytoma is composed of mutated astrocytes uniformly infiltrating around the brain tissues. The tumor cells show less nuclear atypia and slower proliferation. **B** The mass, in the temporal lobe, has isosignal and low intensity on T1-weighted imaging and shows mixed high and low signals on T2-weighted imaging. **C** Anaplastic astrocytomas show nuclear atypia and greater proliferative capacity, with nearby erythrocyte infiltration. **D** The occupied mass, in the temporal lobe, shows low intensity on T1-weighted imaging and high signals on T2-weighted imaging with irregular margins. **E** GBM shows apparent nuclear atypia and prodigious proliferation. **F** The tumor is located in the parietal lobe, with annular high intensity and central low intensity on T1-weighted imaging and mixed signals and peripheral edema signal on T2-weighted imaging. Tumor cell is dividing and duplicating their chromosomes (showing with arrows). Tumor cells masquerade as endothelial cells (showing in boxes). H&E, ×200, bar = 100 microns. GBM Glioblastoma multiforme.

Gliomas are the most common type and highly heterogeneous brain tumors with high morbidity and mortality, constituting 80% of malignant brain tumors [1]. According to World Health Organization (WHO) criteria, gliomas are assigned malignancy grades from circumscribed type I to diffusely infiltrating type II–IV, depending on their histopathological and genetic characteristics [2]. Glioblastoma multiforme (GBM) is a grade IV glioma, representing the most common and lethal sub-type (Fig. 1). Currently, surgical resection, chemotherapy combined with radiotherapy are the modalities of standard GBM treatments and can improve the prognosis of low-grade gliomas to a certain extent [3]. However, due to high-frequency recurrence and eventual death caused by the highly invasive nature and the special anatomical location, the clinical effect of malignant glioma is still not satisfactory.

**Fig. 2:**
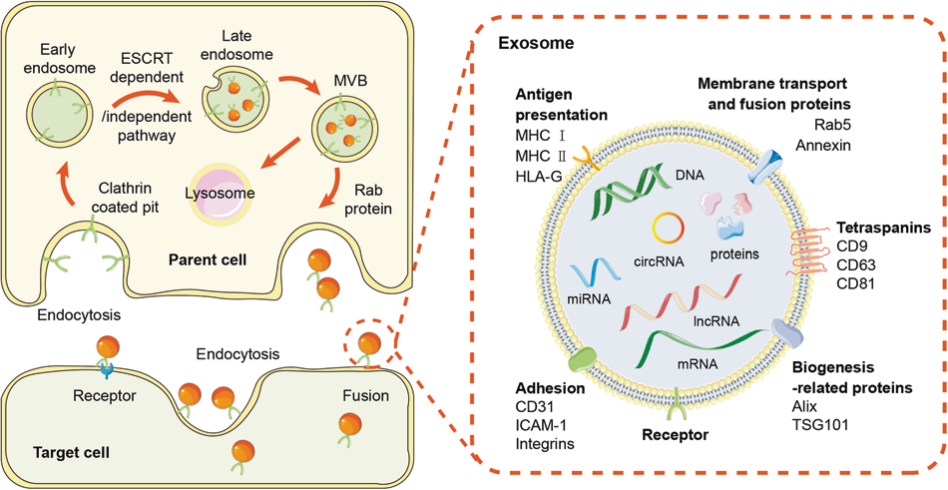
The biogenesis and release of exosomes. Exosome biogenesis begins with early endosome formation through endocytosis at the plasma membrane. The membrane receptors are internalized through clathrin-coated pits and delivered to early endosomes. By ESCRT dependent or independent pathway, the endosomal membrane buds inward to form exosomes, and then early endosomes mature into MVBs. Through this process, exosomes contain a variety of factors including DNA, RNA, and intracellular proteins. Exosomes in MVBs are delivered to lysosomes for degradation or fused with the plasma membrane via the Rab proteins for release to the extracellular space. After secretion, the recipient cell mediates uptake of exosomes through endocytosis, fusion with the plasma membrane, or ligand/receptor interaction. ESCRT Endosomal sorting complexes required for transport, MVB multivesicular body.

**Fig. 3:**
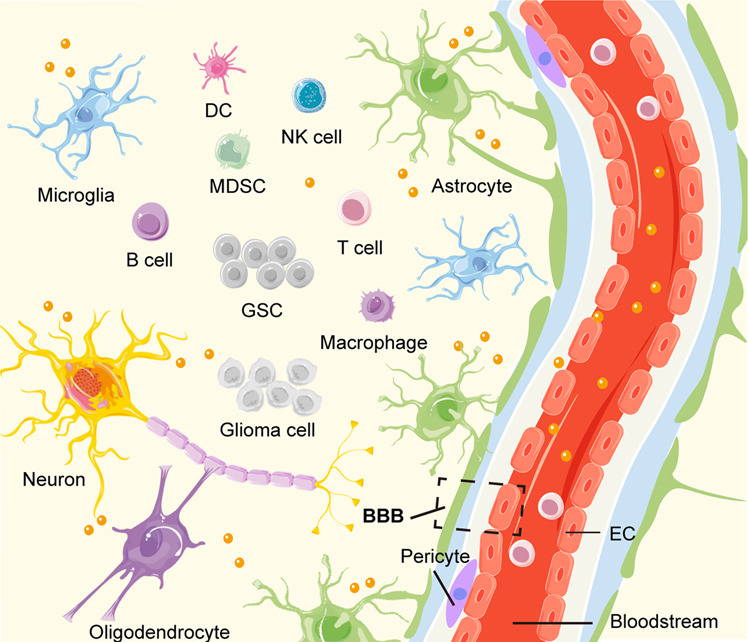
GDEs and multiple cell types in glioma TME. There are many types of cells in the TME, roughly divided into four parts: (1) Tumor cells: glioma cell, GSC; (2) nerve cells: astrocyte, microglia, neuron, oligodendrocyte; (3) immune cells: macrophage, MDSC, DC, T cell, B cell, NK cell; (4) stroma cells: EC, pericyte. Especially, the GDEs mediate the intercellular communication in TME and can cross the BBB, which are expected to be novel targets for the treatment and diagnosis of glioma. GDE Glioma-derived exosome, TME tumor microenvironment, GSC glioma stem cell, MDSC myeloid-derived suppressor cell, DC dendritic cell, NK natural killer, EC endothelial cell, BBB blood-brain barrier.

Generally speaking, exosomes are defined as the small bilayer membrane vesicles with a diameter of 40–160 nm that are derived from endosomes into the extracellular space [4]. Exosomes are composed of multiple conserved tetraspanins (CD9, CD63, CD81), biogenesis-related proteins (Alix and Tsg101), heat shock proteins (Hsp70 and Hsp90), transport proteins (GTPases, annexins, and flotillin), and integrins [5]. Exosome-packed cargoes contain a great diversity of proteins, lipids, DNAs, mRNAs, non-coding RNAs (ncRNAs) (Fig. 2). Various cell types, including neurons, astrocytes, microglia, fibroblasts, endothelial cells (ECs), and other immune cells, constitute the complicated tumor microenvironment (TME) in glioma. Exosomes, especially glioma-derived exosomes (GDEs), have been associated with the tumorigenesis, proliferation, colonization, and metastasis of glioma [6] (Fig. 3).

The functionally important types of ncRNAs in human diseases usually refer to microRNAs (miRNAs), long non-coding RNAs (lncRNAs), and circular RNAs (circRNAs) [7, 8]. MiRNAs consist of about 18–22 bp and mainly function in post-transcriptional repression of targeted gene expression and RNA silencing [9]. MiRNAs are the most well-studied ncRNAs and the abnormal miRNA profile favors glioma growth and invasion. LncRNAs are a class of linear RNA over 200 bp in length without the capability to encode proteins but can regulate gene expression [10]. Multiple lncRNAs have been investigated to be involved in the initiation and progression of glioma including CRNDE, H19, XIST, GAS, Malat1, HOTAIR, and SOX2OT [11]. CircRNAs are covalently closed single-chain circular molecules, which are endogenous molecules that exhibit diversity, evolutionary conservation, relative stability, and specificity [12]. CircRNAs usually function as tumor promoters or suppressors by acting as miRNA sponges to subsequently regulate transcription and splicing of their parental genes. The lncRNA- and circRNA-miRNA-mRNA interaction networks might play vital roles in the modification of glioma progression and therapy resistance [13].

A broad range of ncRNAs encapsulated in GDEs is engaged in fueling the driver mutations and epigenetic modifications in various pathophysiological processes of glioma [14]. In the process of migration through brain parenchyma, glioma cells can release these exosomal gene modifiers as extra-tumoral stimuli to re-shape the premetastatic state. In addition, ncRNAs can be selectively packaged in exosomes to spread into local TME and systemic bio-fluids, with the capability of crossing the blood-brain barrier (BBB) [15]. For the critical clinical management of glioma, GDE ncRNAs are potential accurate, non-invasive, and rapid diagnostic markers in monitoring tumor progression and treatment response.

Given the complex molecular mechanism and expanding research field on GDEs, in this review, we summarize the latest findings on the fundamental roles of GDE ncRNAs that have been implicated in the glioma behaviors, immunological regulation, diagnosis potential, and treatment resistance, as well as the research limitations and perspectives.

## GDE ncRNAs in remodeling glioma behaviors

GDE ncRNAs have yielded confounding influence on glioma progress due to their critical roles in cellular communication [16]. Importantly, GDE ncRNAs can be nonrandomly absorbed by heterologous and homologous cells to affect post-transcriptional genetic regulation, leading to behavioral changes characterized by tumorigenesis, tumor growth, invasion, and metastasis [17] (Table 1).

**Table 1:** Representative tumor-derived exosomal ncRNAs in glioma and their related mechanisms.

NcRNAs	Dysregulation	Exosome isolation techniques	Mechanism	Clinical significance
miR-376c-3p	Upregulated in U251 and U87 cells	Ultracentrifugation	Promoted the progress of GBM via the GOLPH3–miR-376c-3p axis	A new candidate for the treatment of GBM [16]
miR-148a	Upregulated in serum	Precipitation method	Promoted GBM cell proliferation and migration by harboring CADM1 to activate the STAT3 pathway	A predictor and therapeutic biomarker [17]
miR- 221	Upregulated in U87MG cells	Precipitation method	Promoted the SHG-44 cell proliferation, migration, and TMZ resistance via the RELA–miR-221–DNM3 axis	A diagnostic and therapeutic target [18]
miR-301a	Upregulated in serum	Precipitation method	Promoted the proliferation and invasion of glioma-derived H4 cells by downregulating PTEN to activate the AKT and FAK signaling pathways	A diagnostic and prognostic biomarker [19]
miR-454-3p	Downregulated in serum	Precipitation method	Inhibited cell proliferation, migration, invasion, and autophagy in U251 and U87 cells by targeting ATG12	A tumor suppress and an exosomal biomarker [20]
lncRNA LINC00470	Upregulated in serum	Precipitation method	Suppressed autophagy and strengthened the proliferation in U251 and SWO-38 cells, and glioma mouse models via binding to miR-580-3p to modify WEE1 expression and activating the PI3K/AKT/mTOR pathway	A potential therapeutic target [21]
lncRNA ROR1-AS1	Upregulated in tissues	Ultracentrifugation	Promoted cell proliferation and progression in SHG-44 and U251 cells, and xenograft nude mice model via harboring miR-4686	A potential therapeutic target [22]
circMMP1	Upregulated in serum	Precipitation method	Induced the proliferation and motility and inhibited the apoptosis in U251 and LN229 cells, and Xenograft mouse model via the circMMP1–miR-433–HMGB3 axis	A diagnostic and therapeutic target [23]
miR-9	Upregulated in tissues	Ultracentrifugation	Enhanced proliferation, metastasis, and invasion of HUVEC cells via degradation of direct downstream targets COL18A1, THBS2, PTCH1 and PHD3	A potential therapeutic target [29]
miR-21	Upregulated in GSCs	Ultracentrifugation	Promoted the angiogenic ability of ECs by stimulating miR-21/VEGF/VEGFR2 signal pathway	A novel therapeutic target [30]
miR-26a	Upregulated in both tissues and GSCs	Precipitation method	Promoted proliferation, migration, tube formation, and angiogenesis of HBMECs via activating the PI3K/Akt pathway to target PTEN in vitro	A new therapeutic target [31]
lncRNA LncCCAT2	Upregulated in U87MG cells	Ultracentrifugation	Strengthened migration, proliferation, tubular-like structure formation in vitro and arteriole formation in vivo, accompanied by upregulated VEGFA and TGFβ expression by LncCCAT2 entering into HUVECs.	A putative therapeutic target [32]
lncRNA-ATB	Upregulated in A172 and U251 cells	Precipitation method	Accelerated the migration and invasion of glioma cells by the inhibition of miR-204-3p in an Argonaute 2-dependent manner to activate recipient astrocytes	A therapeutic target for the treatment of invasive glioma [37]
miR-199a-3p	Upregulated in tissues and C6 cells	Ultracentrifugation	Upregulated by the HIF-1α activation, and then aggravated oxygen-glucose deprivation and the ischemic injury of HT22 cells via repressing mTOR pathway.	A potential therapeutic target [38]
miR-10a	Upregulated in U87, P3, GL261, and G422 cells	Ultracentrifugation	Mediated GDE-induced MDSC expansion and activation via targeting RORA	A target for MDSCs-based therapy [47]
miR-21	Upregulated in U87, P3, GL261, and G422 cells	Ultracentrifugation	Mediated GDE-induced MDSC expansion and activation via targeting PTEN	A target for MDSCs-based therapy [47]
miR-29a	Upregulated in GL261, G422, U87, and P3	Ultracentrifugation	Intensified the differentiation and propagation of functional MDSCs by targeting Hbp1	A target for MDSCs-based immunotherapy [48]
miR-92a	Upregulated in GL261, G422, U87, and P3	Ultracentrifugation	Intensified the differentiation and propagation of functional MDSCs by targeting Prkar1α	A target for MDSCs-based immunotherapy [48]
miR-1246	Upregulated in U87MG and U251 cells	Ultracentrifugation	Mediated H-GDE-induced M2 macrophage polarization via targeting TERF2IP to activate the STAT3 and inhibit the NF-κB signaling pathway	A novel diagnostic biomarker for GBM and a potential therapeutic target for antitumor immunotherapy [49]
miR-21	Upregulated in BMDM	Ultracentrifugation	Promoted proliferation, migration, and invasion as well as inhibit apoptosis of U87 cells by reducing PEG3	Helpful for the diagnosis and treatment of glioma [50]

*GBM* glioblastoma multiforme, *NcRNA* non-coding RNA, *LncRNA* long non-coding RNA, *miRNA* microRNA, *HUVEC* human umbilical vein endothelial cell, *EC* endothelial cell, *GSC* glioma stem cell, *HBMEC* human brain microvascular endothelial cell, *GDE* glioma-derived exosome, *MDSC* myeloid-derived suppressor cell, *BMDM* bone marrow-derived macrophage.

### GDE ncRNAs in glioma growth and metastasis

The over-expression of Golgi protein GOLPH3 significantly induced 149 differentially expressed (DE) miRNAs in GDEs [18]. GOLPH3 did not affect the content of exosomes, but altered the miRNA profile in GDEs accompanied with the highest increased miR-376c-3p, thus playing a role in the development of GBM. Circulating exosomal miR-148a levels were significantly higher in serum from GBM patients compared with healthy volunteers [19]. MiR-148a delivered by exosomes might promote GBM cell proliferation and migration by harboring CADM1 to activate the STAT3 pathway, indicating the therapeutic biomarker role of exosomal miR-148a in GBM patients. In SHG-44 glioma cells, miR-221 downregulation inhibited cell proliferation and migration and temozolomide (TMZ) resistance, while stimulation with U87MG-derived exosomes played a tumor-promoting role [20]. Lan et al. reported that miR-301a extracted from serum exosomes of GBM patients was bioactive to promote proliferation and invasion of glioma-derived H4 cells [21]. The exosomal miR-454-3p could inhibit cell proliferation, migration, invasion, and autophagy in glioma tissue by targeting ATG12 [22]. Thus miR-454-3p functioned as a tumor suppressor in glioma and might serve as an exosomal biomarker.

In addition, LINC00470 was overexpressed in GDEs and associated with disease severity and postoperative survival time of glioma patients [23]. LINC00470 in GDE could competitively bind to miR-580-3p in glioma cells to modify WEE1 expression and activate the PI3K/AKT/mTOR pathway, thus suppressing autophagy and strengthening the proliferation of glioma cells [23]. LncRNA ROR1-AS1 was upregulated in glioma tissues, and the high level of ROR1-AS1 predicted a poor prognosis [24]. Exosomal ROR1-AS1 induced the progression of glioma cell lines SHG-44 and U251 via harboring miR-4686, which could be a hopeful therapeutic target for glioma clinical treatment. Notably, lncRNA ROR1-AS1 was upregulated in GBM tissues and was associated with a poor prognosis in GBM patients [24]. Intriguingly, lncRNA ROR1-AS1 was encapsulated into tumor exosomes and then promoted SHG-44 and U251 glioma cell proliferation and progression via sponging the miR-4686 both in vitro and in vivo [24]. High serum exosomal circMMP1 level was associated with poor prognosis of glioma patients [25]. CircMMP1 induced proliferation and motility and inhibited the apoptosis of glioma cells by harboring miR-433 to enhance HMGB3 level, thus posing circMMP1/miR-433/HMGB3 axis for glioma therapy [25].

### GDE ncRNAs in angiogenesis

The formation of hypoxic regions in the growing mass of glioma and consequent induction of pro-angiogenic switch are crucial steps in tumor progression [26]. Active angiogenesis is an essential prerequisite for glioma survival and provides conditions ideal for the infiltration and migration of tumor cells at distant niches [27]. Consequently, angiogenesis has been deemed to be a key event and a hallmark in GBM. GDEs contain a variety of functional factors, such as pro-angiogenic and anti-angiogenic, ncRNAs, extracellular proteases that contribute to the induction of proliferation, migration, differentiation, and organization into new tubular structures of the brain microvascular ECs [28].

Metastasis is a multi-step process including the spread of tumor cells from the primary tumor site, the transvascular migration, and the colonization of tumor cells in distant organs [29]. MiR-9 was originally reported to be positively correlated with neurogenesis and was controversial in different tumor types, such as breast cancer and melanoma [30]. MiR-9 was frequently upregulated in glioma specimens and cells, and could enhance proliferation, metastasis, and invasion of glioma cells [31]. GDE miR-9 could be absorbed by vascular ECs, leading to angiogenesis via degradation of direct downstream targets COL18A1, THBS2, PTCH1, and PHD3. Glioma stem cell (GSC)-exosomes could promote the angiogenic ability of ECs by stimulating the miR-21/VEGF/VEGFR2 signal pathway [32]. Wang et al. verified that phosphatase and tensin homolog (PTEN) was downregulated, while miR-26a was upregulated in glioma [33]. The miR-26a overexpressing exosomes in GSCs activated PI3K/Akt pathway via targeting PTEN in vitro, thereby promoting the proliferation and angiogenesis of human brain microvascular endothelial cells (HBMECs). The U87MG cell-derived lncCCAT2 could be absorbed by human umbilical vein endothelial cells (HUVECs) and strongly strengthened migration, proliferation, tubular-like structure formation in vitro, and arteriole formation in vivo, accompanied by upregulated VEGFA and TGFβ expression [34]. As a significant marker of tumor blood vessel abundance, angiogenesis is crucial for the growth and metastasis of glioma by ensuring the sustained nutrients and oxygen supply to abnormal proliferation and expansion of glioma cells. The above evidence shows that exosome-delivered miR-9, miR-26a, and lncCCAT2 play a regulatory role in angiogenesis in the glioma microenvironment. Hence, a further understanding of the mechanism of ncRNAs in glioma angiogenesis will contribute to the development of novel anti-angiogenesis treatments.

### GDE ncRNAs in reshaping neurocytes

GDEs are involved in glioma-neurocyte cross-talk that is an important bidirectional communication associated with dysfunctional homeostasis of glioma [35]. GDEs could be absorbed by various cells of the brain microenvironment, including astrocytes, microglia, and microvascular cells, resulting in tumor-promoting phenotypic change and supporting the tumor progression or recurrence. GDEs-mediated glioma-astrocyte interaction promotes the transformation of astrocytes via metabolic reprograming and implicates horizontal ncRNA transfer in TME remodeling [36].

For instance, Yu et al. showed that O6-alkylguanine DNA alkyltransferase (MGMT)-negative glioma cells could take up reactive astrocyte exosomes and obtain a TMZ-resistant phenotype via translation of exogenous exosomal MGMT mRNA both in vitro and in vivo [37]. Exosome-mediated interaction between astrocyte and glioma conferred a chemoresistance property for the relapse of glioma. Sharma et al. further investigated that GDEs were the potential driving force in promoting the differentiation of rNSCs to astrocytes [38]. GDEs induced the astrocytes activation and could shuttle lncRNA-ATB to astrocytes [39]. The shuttled lncRNA-ATB activated recipient astrocytes by inhibiting miR-204-3p in an Argonaute 2-dependent manner and accelerated glioma migration and invasion. Peritumoral hypoxia in solid tumors is characterized by rapid tumor growth that exceeds vascular supply capabilities and/or tumor vasculature malfunctioning. The growth of HT22 cells was restrained by C6 glioma cells under hypoxic conditions, presuming that GDEs might be the communication medium between peritumoral neurons and glioma cells [40]. Hypoxia-induced GDE miRNA-199a-3p was upregulated by the HIF-1α activation, and then aggravated oxygen-glucose deprivation and the ischemic injury of peritumoral neurons via repressing the mTOR pathway. GDE ncRNAs mediate the interaction between glioma cells and surrounding non-glioma brain cells to promote tumor evolution in the tumor microenvironment. These results shed light on the underlying mechanism of glioma aggressiveness and may provide new targets based on exosome-mediated peritumoral neuronal or neuroglial signals for combating glioma.

## Exosomal ncRNAs in immunological regulation

There is considerable evidence that GDEs contribute to yielding a locally and systemically immunosuppressive state and response to immune-based and therapeutic agents in glioma patients, thus enabling the initiation and progression of neoplasm by evading the antitumor immune response [41]. Deciphering the mechanisms of GDE-mediated immunosuppression is critical for restoring immunosuppressed cell function, including changes in GDE ncRNA content and abundance, intracellular or extracellular pathways for transfer/recognition of antigenic substances and membranes [42].

The differentiation and activation of CD8^+^ T cells are a signature for antitumor immunity response. GDEs could deactivate T cells and hinder lymphocyte migration at high concentrations, but showing an activated phenotype at low concentrations [42]. Tumor-derived exosomes acted as chemorepellents for activated T cells determined in a concentration-dependent way. GSC-derived exosomes could be internalized by monocytes and stimulate the proliferation of PBMCs [43]. Furthermore, both GSCs and exosomes from GBM blood promoted peripheral T cell immunosuppression by acting on PBMC cells to skewing them toward a monocytic myeloid-derived suppressor cell (MDSC) tumor-supportive phenotype. Bu et al. found that dendritic cells (DCs) pulsed with GDEs activated glioma-specific CD8^+^ CTLs from PBMCs to perform recognition and vigorous cytotoxicity to autologous glioma cells [44]. However, Iorgulescu et al. held an inconsistent opinion [45]. GDEs were unable to recapitulate the antigen-presentation machinery, surface co-modulatory signals, or immunosuppressive mediator status of parent cells. They concluded that GDEs played a limited direct role in initiating peripheral immunosuppression. That could be for the differences caused by different experimental conditions, and GDE-carried antigens probably are may first need to be processed by antigen-presenting cells (APCs) to properly elicit antigen-specific immune responses. Exosomes from DCs loaded with glioma cell-derived chaperone-rich cell lysates (CRCLs) boosted powerful and effective antitumor T cell immune response to prolong the survival of GBM-bearing mice.

Immune regulatory cells mainly include regulatory T cells (Tregs) and regulatory B cells (Bregs), MDSCs, and alternatively activated macrophages (M2 type), which are crucial in mediating the immunosuppressive environment and assisting tumors in escaping host immune response [46]. Naive B cells captured the exosomal PIGF to differentiate into TGF-β-positive Bregs, and then further suppressed glioma-specific CD8^+^ T cell activities by inhibiting the CD8^+^ T cell proliferation and the release of perforin and granzyme B [47]. MDSCs represent a major cell population targeted by GDEs, and GDEs could be responsible for the MDSC expansion and activation of their immunosuppressive functions [48]. Hypoxia-stimulated GDEs had a stronger ability to induce MDSCs than normal-GDEs [49]. The hypoxia-induced miR-10a and miR-21 were expressed in GDEs and mediated GDE-induced MDSC expansion and activation by targeting RORA and PTEN. In addition, GDEs were able to intensify the differentiation and propagation of functional MDSCs. Exosomal miR-29a/miR-92a mediated this process by targeting Hbp1, Prkar1α, respectively [50].

Compared with normoxic GDEs, hypoxic GDEs markedly induced M2 macrophage polarization and subsequently promoted glioma proliferation, migration, and invasion in vitro and in vivo [51]. Notably, the most enriched miRNA in hypoxic GDEs and also in the cerebrospinal fluid (CSF) of GBM patients was miR-1246, which mediated hypoxic GDE-induced M2 macrophage polarization by targeting TERF2IP. It inferred that miR-1246 in the CSF might be a novel diagnostic biomarker, a potential target for immunotherapy in GBM. Of cause, the immunosuppressive environment in glioma is a complex entity with multiple cellular reciprocities. Exosomes of non-tumor cell origin are also able to transport endogenous components to tumor cells in favor of glioma immune escape. The induced M2 bone marrow-derived macrophage (BMDM) from healthy peripheral blood could shuffle miR-21 to promote proliferation, migration, and invasion of glioma cells by reducing PEG3 [52].

Exosome-mediated soluble factors can penetrate the BBB and reprogram adjacent and distant immune effects into immunosuppressive phenotypes. These results indicate the irreplaceable functions of GDE ncRNAs, such as miR-29a/miR-92a, miR-1246, miR-21, in regulating CD8^+^ CTL, Tregs, and M2, suggesting a prominent role of GDEs in evading immune surveillance and ultimately leading to altered immune intensity. Therefore, GDE ncRNAs are promising candidates to modulate effective antitumor immune response for targeting constraints of glioma.

## Exosomal ncRNAs as promising diagnostic biomarkers

Classical histological analysis of biopsy specimens is not desirable because of surgical considerations, and may not fully represent all the genetic diversity of GBM cells [53]. Notably, exosomes are of the capability to pass through the intact BBB and anatomical compartments through transcytosis, and then enter the circulatory system [54]. These circulating exosomes containing specific ncRNAs are stable, abundant, reproducible, and disease-specific, overcoming the bottleneck of tissue-specific miRNAs only being detectable in tissues, thus forming the basis for GBM liquid biopsies [55].

MiRNAs have attracted widespread attention as diagnostic and prognostic biomarkers of glioma. In a cohort of glioma patients before and after radiotherapy, Li et al. detected miRNAs extracted from serum exosomes by miRNA sequencing and found 18 upregulated DE miRNAs and 16 downregulated DE miRNAs [56]. It was very interesting that miR-454-3p was prominently downregulated in glioma tissues but significantly upregulated in serum exosomes from the same glioma patients [22]. The expression of miR-21-5p was increased in GBM tissue compared with lower-grade glioma and normal brain tissue, while both miR-9-5p and miR-124-3p were overexpressed in exosomes of GBM stem cell lines [57]. The high exosomal miR-454-3p expression or low tissue miR-454-3p expression was associated with poor prognosis. The level of tumor miR-181d was positively correlated with the functional parameters of glioma patients, while the increased level of exosome miR-181b showed a worse functional outcome [58]. Besides, elevated expression of exosomal miR-181b could manifest a dramatically shortened postoperative survival in GBM patients and was related to patient functions and tumor-related symptoms. Serum miR-301a was overexpressed in gliomas and its expression level increased with ascending grades [21]. Moreover, after surgical removal of the primary tumor, the serum exosome miR-301a level was significantly reduced and increased again when GBM recurred [21].

Since these alterations are either associated with specific stages of glioma or caused due to the therapeutic scheme, the variation of circulating exosomes in the content or expression in glioma patients may be indicative of cancer status. Chun et al. found that human chorionic gonadotropin (hCG) and annexin A5 were changed under temperature stress [59]. These altered proteins or RNAs might be used as signatures for GBM cells when responding to an external stimulus. Wang et al. screened that a total of 109 upregulated and 61 downregulated miRNAs were DE miRNAs in serum exosomes between the patients with intracranial lymphoma and high-grade glioma [60]. Among them, the representative downregulated miR-766-5p and miR-376b-5p were auxiliary diagnostic indicators for high-grade glioma and intracranial lymphoma, and miR-766-5p might be used as a differential diagnostic marker for both diseases. Santangelo et al. observed that the expression levels of miR-21, miR-222, and miR-124-3p in serum exosomes of high-grade gliomas were significantly higher than those of low-grade gliomas and healthy controls, and were sharply decreased in samples obtained after surgery [61]. HOTAIR levels in GBM serum samples were significantly higher than controls and were significantly associated with high-grade brain tumors [62]. The serum-derived exosomes contained HOTAIR could be used as a novel prognostic and diagnostic biomarker for GBM.

Intriguingly, specific protein and mRNA for immune-related genes in plasma exosomes are potential candidates in evaluating glioma patient response to vaccination therapy. Muller et al. isolated exosomes from pre/post-vaccine plasma specimens and found that exosomal IL-8 and TGF-β mRNA positively correlated with post-vaccine immunologic responses, while PD-1 mRNA was persistently upregulated, showing the potential of serving as surrogate markers for monitoring [63]. The inhibitor of apoptosis protein survivin (SVN) promoted cancer cell proliferation, local immune suppression, and resistance to chemotherapy and it is a potential cancer biomarker [64]. In the malignant glioma patients receiving the anti-SVN vaccine, the CD9^+^/GFAP^+^/SVN^+^ and CD9^+^/SVN^+^ exosomes were released into the circulation while their early reduction after anti-SVN immunotherapy was related to longer progression-free survival [64]. These studies confirmed the feasibility of GDE ncRNAs in individualized treatment monitoring.

Advances in microarrays and other experimental techniques have made it possible to detect abnormal ncRNA expression patterns in gliomas that are associated with specific tumor stages, metastasis, low survival, disease outcome, and response to specific therapies. The existing studies have emphasized the potential and possibility of GDE ncRNAs in various aspects of differential diagnosis, prognosis diagnosis, staging and grading characteristics, and evaluation of vaccine therapy. At present, effective non-invasive diagnostic indicators and methods for identifying different grades of glioma are still limited, but ncRNAs in GDEs provide new candidate biomarkers for differential diagnosis, monitoring progression, evaluating prognosis (Table 2). Synergistically adding the epigenetic information to routine examinations, including MRI imaging, MGMT promoter methylation, IDH mutation, loss of 1p/19q, BRAF fusion, and CpG island methylator phenotype (CIMP), are of crucial and predictive significance for glioma diagnosis.

**Table 2:** The value of tumor-derived exosomal ncRNAs in glioma diagnosis.

NcRNAs	Dysregulation	Exosome isolation techniques	Diagnosis value
miR-454-3p	Downregulated in glioma tissues and upregulated in serum exosomes	Precipitation method	Associated with poor prognosis [20]
miR-21-5p	Upregulated in glioma tissues	Ultracentrifugation	Associated with poor prognosis [55]
miR-9-5p	Upregulated in exosomes of GBM stem cell lines	Ultracentrifugation	Associated with poor prognosis [55]
miR-124-3p	Upregulated in exosomes of GBM stem cell lines	Ultracentrifugation	Associated with poor prognosis [55]
miR-454-3p	Downregulated in glioma tissues and upregulated in exosomes of GBM stem cell lines	Ultracentrifugation	Associated with poor prognosis [55]
miR-181d	Downregulated in glioma tissues and upregulated in serum exosomes	Precipitation method	Showed a worse functional outcome and shortening postoperative survival in GBM patients [56]
miR-301a	Upregulated in serum exosomes	Precipitation method	Reflected the glioma-bearing status and being an independent prognostic parameter for overall survival in advanced grade patients [19]
miR-766-5p	Downregulated in serum exosomes	Precipitation method	An auxiliary diagnostic marker for high-grade glioma and intracranial lymphoma [58]
miR-376b-5p	Downregulated in serum exosomes	Precipitation method	An auxiliary diagnostic marker for high-grade glioma and intracranial lymphoma [58]
miR-21	Upregulated in serum exosomes	Precipitation method	A minimally invasive biomarker for differential diagnosis of gliomas and preoperative prediction of glioma grade before surgery [59]
miR-222	Upregulated in serum exosomes	Precipitation method	A minimally invasive biomarker for differential diagnosis of gliomas and preoperative prediction of glioma grade before surgery [59]
miR-124-3p	Upregulated in serum exosomes	Precipitation method	A minimally invasive biomarker for differential diagnosis of gliomas and preoperative prediction of glioma grade before surgery [59]
lncRNA HOTAIR	Upregulated in serum exosomes	Ultracentrifugation	Associated with high-grade brain tumors [60]
IL-8 mRNA	Upregulated in plasma	Ultracentrifugation	Positively correlated with post-vaccine immunologic responses [61]
TGF-β mRNA	Upregulated in plasma	Ultracentrifugation	Positively correlated with post-vaccine immunologic responses [61]
PD-1 mRNA	Upregulated in plasma	Ultracentrifugation	Potentially served as surrogate markers for monitoring [61]

*GBM* glioblastoma multiforme, *NcRNA* non-coding RNA, *GSC* glioma stem cell, *LncRNA* long non-coding RNA.

## GDE ncRNAs in treatment resistance

GBM tumors exhibit a large amount of genetic heterogeneity at the inter-tumor and intra-tumor levels, which makes the characterization establishment and standardized treatment more challenging [65]. As a monofunctional oral DNA-alkylating antitumor agent, TMZ is commonly used as first-line chemotherapy in glioma by inducing DNA damage of glioma cells [66]. Many glioma patients who received TMZ developed chemotherapy resistance and often possess a hypermutation phenotype evolution, partly owing to the elevated MGMT and lack of DNA repair pathways [67]. GDEs can horizontally propagate TMZ chemotherapy resistance in recipient cells, which may involve the transfer of resistance-induced molecules, such as P-glycoprotein (P-gp), mRNAs, miRNAs, lncRNAs, and circRNAs [68].

GDE ncRNAs harbor great values to modulate radiation resistance and chemoresistance in the sensitive recipient cells. GDE miR-301a was hypoxia-associated and was a compelling target for enhancing the radiation resistance effect of GBM [69]. Exosomal miR-301a induced by hypoxic GBM cells transferred to adjacent cell types to foster radiation resistance, by inhibiting TCEAL7 expression and the Wnt/β-catenin pathway. The ability of GBM cells to survive radiation was occurred by cellular communication mediated via exosomes [70]. Radiation-induced exosomes increased tumor burden, whereas heparin and simvastatin inhibited resistant/proliferative capacities by uptake suppression of exosomes in recipient cells [70]. Furthermore, radiation-induced exosomes contained increased oncogenic miR-889, mRNAs, and proteins of the proteasome pathway, which were in favor of resistant/proliferative profiles.

In clinical tissue samples, exosomal miR-221 expression was elevated positively with glioma grades [20]. Especially, the exosomal miR-221 targeted DNM3 to induce glioma progression and TMZ resistance [20]. In TMZ-resistant glioma cells, circ-HIPK3 was upregulated, while miR-421 was significantly downregulated in TMZ-resistant glioma exosomes [71]. Interestingly, exosomal circ-HIPK3 facilitated tumor progression and TMZ resistance by directly modulating the miR-421/ZIC5 axis. In the serum of TMZ-resistant patients, exosomal circNFIX was upregulated and indicated a poor prognosis [72]. Exosomal circNFIX derived from TMZ-resistant cells sponged miR-132 to strengthen cell growth under TMZ exposure, thus endowing recipient cells with TMZ resistance.

LncRNA SBF2-AS1 could trigger the TMZ resistance, whereas SBF2-AS1 inhibition sensitized the resistant GBM cells to TMZ [73]. SBF2-AS1 could act as a competing endogenous RNA (ceRNA) for miR-151a-3p, leading to the disinhibition of endogenous target XRCC4, which enhanced double-strand break (DSB) repair in GBM cells. It concluded that GBM cells remodeled TME by secreting oncogenic lncSBF2-AS1-enriched exosomes for promoting tumor chemotherapy resistance. HOTAIR was a significantly upregulated lncRNA in TMZ-resistant GBM cells [74]. The inhibition of exosome-mediated HOTAIR transfer was involved in suppressed proliferation, EMT, and TMZ resistance through the miR-519a-3p/RRM1 axis.

Interestingly, not all investigated exosomal ncRNAs are genetic modifiers that lead to enhanced TMZ resistance. For example, Zeng et al. characterized exosomes from TMZ-resistant cell lines, serum, and CSF and determined the effect of exosomes from TMZ-resistant cells on recipient GBM cells [75]. The miR-151a expression sensitized TMZ-resistant GBM cells via inhibiting XRCC4-mediated DNA repair. Restoration of exosomal miR-151a from the parental TMZ-resistant cells eliminated the chemoresistance spread that was transferred by donor TMZ-resistant cells.

Summarily, GDE ncRNA plays a vital biological role in the process of TMZ chemotherapy resistance and radiotherapy in glioma. Generally speaking, GDE-mediated ncRNAs incorporation into TME cells could upregulate tumor activity and drive therapeutic sensitivity **(**Table 3**)**. Totally, GDEs and their ncRNA cargoes are not only essentially monitoring markers that might predict chemotherapy response and therapeutic effects but also represent promising therapeutic targets for intractable GBM independently or complimentary.

**Table 3:** The value of tumor-derived exosomal ncRNAs in glioma treatment resistance.

NcRNAs	Dysregulation	Exosome isolation techniques	Mechanism	Clinical significance
miR-301a	Upregulated in serum exosomes	Precipitation method	Inhibited tumor suppressor TCEAL7 expression and the Wnt/β-catenin signaling pathway	Enhanced the radiation resistance effect of GBM [67]
miR-889	Upregulated in SH-SY5Y, U87, and STS26T cells	Precipitation method	Inhibited DAB2IP expression	Promoted glioma proliferation and radiation resistance [68]
miR-221	Upregulated in U87MG cells	Precipitation method	Targeted DNM3	Induced glioma progression and TMZ resistance [18]
circ-HIPK3	Upregulated in serum exosomes and NHA, A172, U251, A172/TR, and U251/TR cells	Ultracentrifugation	Modulated the miR-421/ZIC5 axis in glioma	Facilitated glioma progression and TMZ resistance [69]
circNFIX	Upregulated in serum exosomes	Precipitation method	Sponged miR-132	Endowed glioma cells with TMZ resistance [70]
lncRNA SBF2-AS1	Upregulated in tissues and U87, LN229, A172, T98, and U251 cells	Precipitation method	Sponged miR-151a-3p, thus leading to the disinhibition of its endogenous target XRCC4, which enhanced DSB repair in GBM cells	Triggered the TMZ resistance [71]
lncRNA HOTAIR	Upregulated in serum exosomes and A172 and LN229 cells	Precipitation method	Regulated the miR-519a-3p/RRM1 axis	Promoted the TMZ resistance [72]
miR-151a	Downregulated in GBM tissues and U251 and N3 cells	Ultracentrifugation	Inhibited XRCC4-mediated DNA repair	Sensitized TMZ-resistant GBM cells [73]

*NcRNA* non-coding RNA, *GBM* glioblastoma multiforme, *TMZ* temozolomide, *LncRNA* long non-coding RNA, *DSB* double-strand break.

## Limitations and perspectives

Despite the suitability of GDE ncRNAs as biomarker reservoirs at the preclinical study level, many unanswered questions and challenges in this field are mentioned regarding GDE ncRNAs’ mechanism, detection, and therapeutic potential.

There are limitations of exosomes in source, isolation, purification, and identification. Quality control is strictly needed for clinical applications, which require a high degree of standardization, involving the isolation of cells, the isolation of cultured serum and exosomes. Identifying and isolating GDEs is particularly important, but may be difficult to rigorously implement at present on account of the pros and cons of the various methods for isolation. GDEs involvement in the induction mechanism of exosome release, selective packaging ability, proteome and genome in encapsulation, and uptake of tumor and non-tumor cells are all important mechanisms to understand the malignant biological characteristics of glioma but are still hazy at the moment. Since exosomes are complicated in composition like biochemical cocktails, it is important to note that the biosafety and effectiveness of exosome-based clinical therapies are somewhat unpredictable due to their compositional complexity.

Secondly, the mechanism of GDEs in glioma is still poorly understood and needs to be further clarified. It is well known that exosomes with other cell types and various factors, such as growth factors, chemokines, and enzymes, constitute a complex TME network with dynamic evolution. Exosomes produced by fibroblasts, astrocytes, and immune cells can carry ncRNAs, proteins, and cytokines that are internalized into different recipient cells to modulate different cellular processes. For instance, Murgoci et al. confirmed that microglia-derived exosomes could suppress tumor invasion in time course tested on a 3D spheroid glioma culture [37]. In a mouse model, neuronal exosomal miR-124-3p could exert genetic regulation of astrocyte functions [76]. Therefore, due to the high heterogeneity of gliomas, it is difficult to determine which cell type-derived exosome and which GDE ncRNA dominates the influence of tumor progress at particular staging and typing of glioma.

Currently, exosome-based non-invasive biomarkers are very attractive for gliomas detection. GDE ncRNAs are originated from glioma cells and are hypersensitive to subtle pathophysiological alterations, manifested by changes in abundance and content. Besides, due to the relatively easy availability, circulation ability, and protection from ncRNA degradation, the GDEs with cargo-specific patterns are more sensitive and specific to predict application compared with other conventional biochemical indicators. In glioma cell lines, HSPs are involved in multiple pathways of cell proliferation, survival, invasion, and migration, and which have been proved to be a constant cargo feature of exosomes [77]. Therefore, GDEs that contain large amounts of HSPs have high diagnostic potential. The ncRNA repertoire of GDE separated from CSF and serum is being developed as a liquid biopsy platform in glioma. However, none of the studied GDE ncRNAs have been introduced into final clinical practice currently. Many GDE ncRNAs studies reflect preclinical level changes rather than ultimate human changes.

Novel therapies, including small-molecule inhibitors, immunotherapeutic agents, chimeric antigen receptor (CAR) T cell therapy, and tumor treating fields, have been introduced to GBM treatment strategies. Among them, as GDEs play a key role in the entire glioma progression, suppression of GDE secretion and clearance strategies targeting GBM-oriented exosomes are gradually being developed. Almost all studied GDEs have shown potential as therapeutic targets to inhibit growth or metastasis through gene intervention regimens. It is promising to exploit the targeted therapy by the manipulation of exosomes to enable the delivery of molecular or pharmacological therapeutics. The engineered exosomes could successfully cross the BBB for the treatment of glioma. But compare to detection potential, the exosome-based therapeutic application needs a long way to go for the clinic in consideration of safety and effect evaluation.

According to the above information, there are immeasurable potentials and perspectives that are being explored based on GDE ncRNAs for future application. At present, it is still intriguing to strive to focus on the epigenetic regulation of GDE ncRNAs in glioma. A better understanding of the interaction mechanism between GDE ncRNAs and TME is helpful to identify potential clinical therapeutic targets. Research on exosomal ncRNAs may completely revolutionize the diagnosis and treatment mode of glioma. The innovative diagnosis and treatment based on exosomal ncRNAs are being propelled by continuous technological advances such as allowing higher resolution of GDE ncRNAs acquisition and accelerating the discovery, design, and optimization of new compounds that can regulate exosomal ncRNAs production and delivery. The clinical application of GDE ncRNAs will be developed in collaboration with complementary diagnostic and therapeutic platforms, such as nanotechnology, immunotherapy, RNA aptamers, microfluidics. Characteristics of ideal diagnostic markers include bioavailability, ease of separation, and the ability to accurately provide significant information about disease status. An interesting question is whether the content and abundance of exosomes will change accordingly during different stages of glioma occurrence and development? It is worth pondering to determine the potential of circulating or tumor tissue-derived GDE ncRNAs used as an independent predictor, or in combination with other diagnostic indicators. Thus, GDE ncRNAs represent a huge promising but not yet fully explored source of reliable biopsy biomarkers complementary to the differential diagnosis, monitoring of post-surgical glioma progression, and even the prediction of the patient response to personalized therapies, such as vaccines, antibody drugs, and chemoradiotherapy.

It is equally important that the strategy of specifically targeting exosomes or their ncRNA cargoes may be valuable treatment options for the treatment of glioma. Similarly, many studies aim to regulate exosome production or block exosome uptake to treat gliomas, providing a new treatment for patients with gliomas. Besides, another actual prospect of GDEs may include the development of novel DC vaccines with advanced properties to induce or promote effective tumor-specific responses. Due to partial breakdown of the blood-brain barrier in glioma and increased leakage caused by disruption of the normal astro-endothelial cell relationship, exosomes can reach the tumor area and deliver cytotoxic drugs. Therefore, GDE is considered to be an effective tool for new targeted anti-cancer drug delivery, but at the same time, there is still a long way to go, as most of these studies are currently in the preclinical stage. Briefly, in the actual clinical transformation process, the GDE therapeutic applications have still faced the difficulties of human dose, administration interval, and administration mode on biosafety and efficacy need more comprehensive data support than single-component drugs. The further investigation and optimization of processes such as isolation, characterization, enrichment, cargo loading, and reconstruction of target specificity to exosomes, are quite important for exosome-based applications for glioma patients. Finally, other issues related to the potential clinical application of exosomes in glioma, including follow-up, ethics, quality control, techniques, and supervision, are also rather challenging.

## Conclusion

**Fig. 4:**
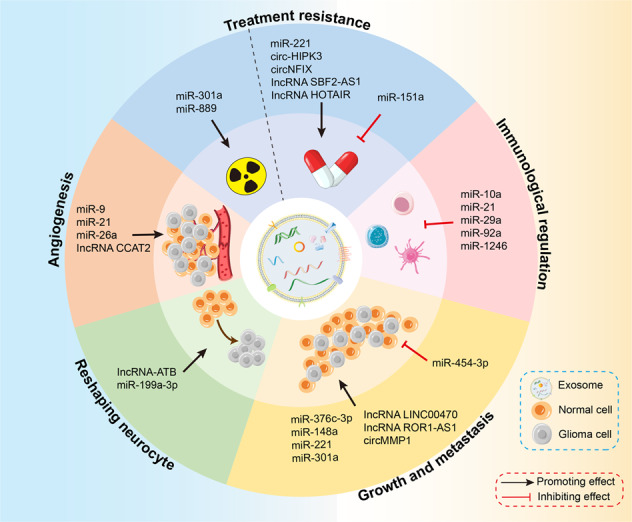
The biological function of exosomal ncRNAs in glioma. The various types ncRNAs specifically present in exosomes largely depend on their cellular origin. GDE ncRNAs play a key role in cell communication, thus mediating a wide range of glioma processes and possessing a confounding effect on glioma progression. More importantly, GDE ncRNAs can be nonrandomly absorbed by heterologous and homologous cells, affecting post-transcriptional gene regulation and leading to behavioral changes characterized by glioma growth and metastasis, angiogenesis, neurocyte reshaping, immune response, treatment resistance. ncRNA Non-coding RNA, GDE glioma-derived exosome.

GDEs contain a variety of functional molecules that can reflect the complex heterogeneity of gliomas. Collectively, GDEs represent a novel means of intercellular cross-talk within the TME by delivering various ncRNAs to affect post-transcriptional genetic regulation, and thus participate in tumor growth, migration, cellular invasion of the surrounding brain, angiogenesis, tumor-derived immune suppression for supporting glioma progression (Fig. 4). Thus, GDEs and GDE-loaded ncRNAs “educate” surrounding homogenous and heterogeneous cells to drive them toward a tumor-promoting phenotype. It is worth mentioning that GDE-derived miR-376c-3p, miR-148a, miR-221, miR-301a, lncRNA LINC00470, and lncRNA ROR1-AS1, circMMP1 are promoting factors while miR-454-3p is the suppressing factor in glioma tumorigenesis, growth, and metastasis. Although not yet exploited in a clinical setting, the GDE studies reviewed here still emphasized the possibilities of their potential in translational medicine. For therapeutic impact, GDE-derived miR-221, miR-151a, circ-HIPK3, circNFIX, lncSBF2-AS1, and HOTAIR are supposed to participate in several types of treatment resistance. Nowadays, there is accumulating evidence regarding the crucial role of GDE ncRNAs in the glioma initiation and course, but this topic is still in its infancy. Lastly, the continuing preclinical validation and clinical exploration of the dysregulation levels and mechanisms of GDE ncRNAs in human gliomas are now warranted to shed light on the development of more effective diagnostic and therapeutic strategies.

## Supplementary information

Declaration of contributions to article

Reproducibility Checklist
