# Supplementary material for: Current landscape of tumor-derived exosomal ncRNAs in glioma progression, detection, and drug resistance
Source: Cell Death Dis. 2021 Dec 9;12(12):1145. doi: 10.1038/s41419-021-04430-z (PMC8660802; doi:10.1038/s41419-021-04430-z)
Supplement: Supplementary file 1 — Declaration of contributions to article [file 41419_2021_4430_MOESM1_ESM.pdf]

DECLARATION OF CONTRIBUTIONS TO ARTICLE

ADMC

Manuscript Number: **CDDIS-21-2420RR**

Journal Name: *Cell Death & Disease* (the 'Journal')

Proposed Title of the Contribution:

Current landscape of tumor-derived exosomal ncRNAs in glioma progression, detection and drug resistance (the 'Contribution)

Author(s):

Xiao He, Yiwei Qi, Xian Zhang, Xiaojin Liu, Xingbo Li, Sihan Li, Yiping Wu, Qi Zhang (the 'Authors')

For all *CDDis* articles, each person named as an author in the published version must be able to show he or she has contributed substantially to the article.

Authorship credit should be based on 1) substantial contributions to conception and design, acquisition of data, or analysis and interpretation of data; 2) drafting the article or revising it critically for important intellectual content; and 3) final approval of the version to be published. Authors should meet conditions 1, 2 and 3.

Any person who cannot be shown to have made a substantial contribution to the article cannot be listed as an author in the final version. The name of any person who is deemed to have made a minor contribution can, however, appear in the Acknowledgments section of the article.

Please complete the table below to indicate the contributions of all named authors to the manuscript.

Author Full Name:	Specification of Contribution to the Manuscript:
Xiao He	investigate the related papers; draft the manuscript; draw related figures
Yiwei Qi	investigate related literature; draw related figures
Xian Zhang	help draft the manuscript
Xiaojin Liu	help revise the manuscript, including figures and tables
Xingbo Li	help revise the manuscript and draw related figures
Sihan Li	help investigate related literature and revise the manuscript
Yiping Wu	design the outline; draft the manuscript; final approve of the version to be published
Qi Zhang	design and revise the outline; final approve of the version to be published

Please complete the table below to indicate the contributions of all named authors to the figures.

Figure 1:

Yiwei Qi provided the MRI images and drafted the figure legend. Xiaojin Liu investigated related literature.

Figure 2:

Xiao He, Xingbo Li investigated related literature, drew the biogenesis and release of exosomes and finished the figure legend.

Figure 3:

Xiao He designed the outline and drew the draft of the figure 3. Sihan Li modified the colors and drafted the related figure legend. Xian Zhang supplemented the contents of the figure.

Figure 4:

Xiao He and Yiwei Qi investigated related literature and designed the outline of the figure 4. Sihan Li supplemented the details and drafted the figure legend

Figure 5:

Figure 6:

Signed for and on behalf of the Author(s):	Print Name:	Date:
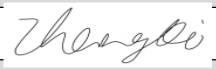	Qi Zhang	November 10th, 2021
